# Glutamic acid intake by formula-fed infants: are acceptable daily intakes appropriate?

**DOI:** 10.1007/s00431-023-05215-6

**Published:** 2023-09-30

**Authors:** Julie A. Mennella, Alissa D. Smethers, Michelle T. Delahanty, Virginia A. Stallings, Jillian C. Trabulsi

**Affiliations:** 1 Monell Chemical Senses Center, Philadelphia, PA 19104-3308 USA; 2 Department of Behavioral Health and Nutrition, University of Delaware, Newark, DE 19173 USA; 3 Children’s Hospital of Philadelphia, Philadelphia, PA; Department of Pediatrics, Perelman School of Medicine at the University of Pennsylvania, Philadelphia, 19146 USA

**Keywords:** Glutamic acid, Monosodium glutamate, Acceptable daily intake, Infant, Formula, Complementary diet, Safety

## Abstract

The 2017 European Food Safety Authority (EFSA) recommendation of an acceptable daily intake (ADI) of 30 mg glutamic acid/kg bw/day did not take into consideration the primary energy sources during infancy, including infant formulas. In the present study, we determined total daily intakes of glutamic acid in a contemporary cohort of healthy infants who were fed either cow milk formula (CMF) or extensive protein hydrolysate formula (EHF); the formulas differed substantially in glutamic acid content. The infants (*n* = 141) were randomized to be fed either CMF or EHF. Dietary intakes were determined from weighed bottle methods and/or prospective diet records, and body weights were measured on 14 occasions from 0.5 to 12.5 months. Secondary data analysis determined the glutamic acid content of the diet over time. The trial was registered at http://www.clinicaltrials.gov/ as NCT01700205, 3 October 2012. Glutamic acid intake from formula and other foods was significantly higher in infants fed EHF when compared to CMF. As glutamic acid intake from formula decreased, intake from other nutritional sources steadily increased from 5.5 months. Regardless of formula type, every infant exceeded the ADI of 30 mg/kg bw/day from 0.5 to 12.5 months.

*Conclusion*: Given that the ADI recommendation was not based on actual intake data of primary energy sources during infancy, the present findings on the growing child’s ingestion of glutamic acid from infant formula and the complementary diet may be of interest when developing future guidelines and communications to parents, clinical care providers, and policy makers.

**Table:** 

**What is Known:** *• The 2017 re-evaluation of the safety of glutamic acid-glutamates and the recommended acceptable daily intake (ADI) of 30 mg/kg bw/d by the European Food Safety Authority (EFSA) did not include actual intake data of the primary energy sources during infancy.*	
**What is New:** *• During the first year, glutamic acid intake from infant formula and other food sources exceeded the ADI of 30 mg/kg bw/day.*

**Supplementary Information:**

The online version contains supplementary material available at 10.1007/s00431-023-05215-6.

## Introduction

Human milk substitutes (herein referred to as *infant formula*) first became commercially available during the late nineteenth century and harmonized laws to ensure their safety and nutritional adequacy first emerged a century later [1]. Assessment of the safety and suitability of ingredients used in infant formulas is an ongoing process as is the evaluation and risk analysis of additives in the food supply for the whole population, including infants and children.

The non-essential, amino acid glutamic acid is found in the diet in free (non-protein bound) and bound form, and one of its salts (monosodium glutamate, MSG) is a well-known flavor enhancer additive. Although considered a Generally Recognized as Safe (GRAS) substance by the US Food and Drug Administration and considered safe by other health organizations, including the Joint FAO/WHO Expert Committee on Food Additives and the European Food Safety Authority (EFSA), the safety of glutamic acid–glutamates as food additives was re-evaluated by the EFSA Panel on Food Additives and Nutrient Sources [2]. The re-evaluation consisted of scientific reviews of chronic neurotoxicity studies in test animals and clinical studies in humans, and an exposure assessment of dietary sources of “glutamic acid–glutamate” with the acknowledgement that the panel could not distinguish that which occurs naturally or was an additive (e.g., MSG) in particular foods or how much was free or bound. In 2017, based on their re-evaluation of test animal neurotoxicity data, EFSA recommended the change from a non-specified acceptable daily intake (ADI) to a group ADI of 30 mg/kg bw/day for glutamic acid, which the panel highlighted was currently being exceeded by all population groups [2]. 

In the years following this EFSA report, many questioned the strength of the evidence and the appropriateness of using a risk assessment paradigm for a macronutrient [3–6]. Particularly relevant for the youngest members of the European Union, the evidence review did not include the primary energy source during infancy, namely human milk and/or infant formula. Not only is glutamic acid the most abundant free amino acid in human milk but it varies greatly among the different types of infant formulas [7]. Based on measured free glutamic acid concentrations in human milk and different types of infant formulas and reference standards of energy intake by 4-month-old infants, Koletzko [5] estimated that while infants who are fed cow milk formulas (CMF) would not exceed the ADI, that was not the case for those fed human milk or extensive protein hydrolysate formulas (EHF). He concluded that setting an ADI below 250 mg/kg bw/day would be inappropriate for healthy infants.

To further extend the analyses put forth by Koletzo [5], here we report on total daily intakes of glutamic acid (free and bound) over the first year in a contemporary cohort of healthy infants who were never breastfed [8]. Intakes were determined from weighed bottle methods and/or prospective diet records and body weights were measured, not estimated. These data highlight the wide range of intakes from the time that formula was the infant’s sole source of energy to when they transition to a diet containing other foods.

## Methods

Healthy term infants with no family history of atopy and whose mothers decided to exclusively formula feed were randomized to be fed either CMF (Enfamil™, Mead Johnson Nutrition; *n* = 59) or EHF (Nutramigen™, Mead Johnson Nutrition; *n* = 54) to investigate the effect of infant formula composition on growth and energy balance from the age of 0.5 to 12.5 months [8]. The formulas were isocaloric (67.7 kcal/100 ml) and provided gratis to the family throughout the trial. The trial was conducted according to the guidelines of the Declaration of Helsinki, approved by the Office of Regulatory Affairs at the University of Pennsylvania Institutional Review Board and registered online at clinicaltrials.gov prior to its start (NCT01700205; 2012–2016). Written informed consent was obtained from each mother prior to study entry.

At each of 14 study visits from 0.5 to 12.5 months, infants were weighed in triplicate by trained research personnel using calibrated infant scales accurate to 0.001 kg and mothers prospectively recorded their infants’ intake of formula and, if applicable, types and amounts of all other liquids and foods. The baseline (0.5 month) visit occurred before randomization (0.75 months) and all infants were fed CMF during the interim. Three-day records were obtained at 0.75, 3.5, and 12.5 months during which formula intake was determined by 3-day weighed bottle intake whereas 1-day diet records were obtained at all other visits. Returned records were reviewed for quality by registered dietitians and deemed not usable if insufficient information (e.g., amounts) provided.

In this secondary analysis, we analyzed the diet records to determine glutamic acid intake from infant formula and other food sources by using the Nutrition Data System for Research (version 2019) software and established food categories [9]; commercial foods marketed for infants were subcoded as baby foods (BF). The glutamic acid content of the two specific brands of formulas was 262.4 mg/100 ml (CMF) and 436.2 mg/100 ml (EHF). From these data, we calculated for each infant the daily intake of glutamic acid (mg/kg bw) and identified its major food sources over time.

### Statistical analysis

**Fig. 1:**
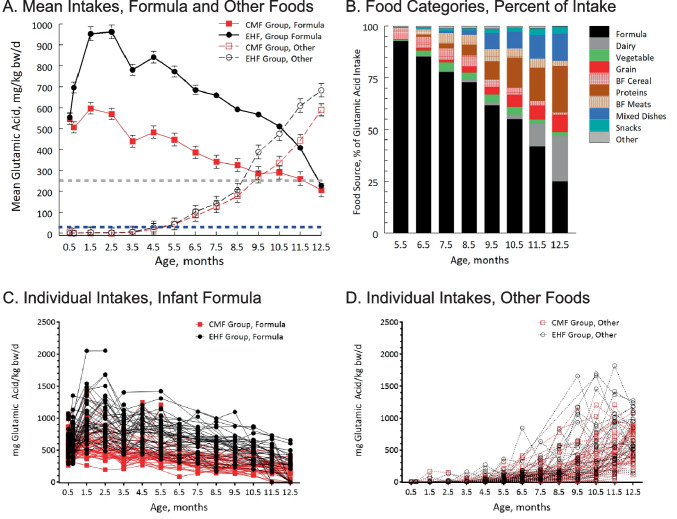
Daily intakes of glutamic acid per kg body weight from infant formula and other foods. **A** Mean formula intakes (± standard error) of infants randomized to be fed either cow milk formula (CMF group, *n* = 59) or extensive protein hydrolysate formula (EHF group, *n* = 54). Slashed blue lines indicate current EFSA Adequate Daily Intake of 30 mg/kg bw/day and slashed grey lines indicate Koletzko’s recommendation of 120 mg/kg bw/day [5]. **B** Percent of total glutamic intake (mg/kg bw/day) from infant formula and major food categories [9]; and **C**, **D** data of each individual infant for infant formula (**C**) and other foods (**D**) intakes. For all, the 0.5-month visit was the baseline visit before randomization, when all infants were fed CMF. Dairy includes milks, yogurt, cheese, ice cream; Grains include breads, pasta, rice, oatmeal, and grain-based desserts; Mixed dishes include meat- and grain-based combination meals including pizza and soups; Proteins include meats, poultry, seafood, eggs, beans/peas, and nuts; Snacks and Sweets include savory snacks, sweet bakery products, crackers, and desserts; Other includes all other food categories. Abbreviation: BF, baby food

We computed descriptive statistics and continuous variables were tested for normality using the Shapiro-Wilk test. To examine whether the glutamic acid intake from infant formula and other foods differed between the randomized groups and over time, we conducted multi-level modelling with generalized estimating equation linear models on intent-to-treat intake data that included randomized group (CMF, EHF) as the between-subjects factor, time (0.5, 0.75, 1.5, 2.5, 3.5, 4.5, 5.5, 6.5, 7.5, 8.5, 9.5, 10.5, 11.5, 12.5 months) as the within-subject factor, and the group × time interaction. To illustrate the sources of glutamic acid in the infants’ diet over time, we computed the percentage of glutamic acid from each of the food category for each infant. We focused on the age range of 5.5–12.5 months since infants were, on average, first introduced to solid foods at 5.3 (± 1.7) months; there was no significant group difference in the age of introduction (*p* = 0.59). Data were analyzed using Stata/IC version 14.2 (Statacorp LLC, College Station, TX) with a significance criterion set at *p* < 0.05, and the glutamic acid intake values were expressed as mean (± standard error of the mean) in Fig. 1A or as mean, median, and upper and lower quartiles in Supplementary Table 1 (online). The data of each individual infant are depicted in Figs. 1C and D; no imputations were made for missing data.

## Results

The infants (50% female) were diverse in maternal-reported race identity (62% Black, 22% White, and 16% Other/More than one race). The study design, inclusion and exclusion criteria, additional subject characteristics, and the trial profile have been published previously [8]. Supplementary Table 2 (online) provides the enrollment numbers at each assessment during trial.

There were significant group (*p* < 0.001) and time (*p* < 0.001) effect on glutamic acid intake from infant formula during the first year, and significant group × time (*p* = 0.01) and time (*p* < 0.001) effects for glutamic acid intake from other foods sources from 5.5 to 12.5 months. Figures 1A presents the group means of the daily intakes of glutamic acid from formula and other food sources, Fig. 1B identifies the top foods sources of glutamic acid in the diet, and Figs. 1C and D plot the intakes of each individual infant from infant formula and other foods, respectively, over time. As expected, the glutamic acid intake data were not normally distributed. Thus, Supplementary Table 1 (online) provides the means, medians, and upper and lower quartiles of glutamic acid intake for each group over time.

Every infant exceeded the ADI of 30 mg/kg bw/day from 0.5 to 12.5 months. The type of formula mattered. Glutamic acid intake from formula was significantly higher in infants fed EHF when compared to CMF. As glutamic acid intake from formula steadily decreased in both groups, intake from other nutritional sources steadily increased from 5.5 months onwards. Figure 1B shows how much infant formula, specially prepared BF cereals and meats and then vegetables, grains, protein foods (e.g., meats, fish), mixed dishes, snacks and sweets, and dairy, contributed to infants’ glutamic acid intake during their first year.

## Discussion

As expected, daily intakes of glutamic acid from infant formula were significantly higher in infants randomized to EHF than CMF. However, when we accounted for other sources of nutrition and used measured rather than estimated intakes, every infant, regardless of the type of formula, exceeded the ADI for glutamic acid–glutamates (30 mg/kg bw/day) recommended by EFSA for all age groups [2]. The vast majority of infants also exceeded the proposed ADI of 250 mg/kg bw/day by Koletzko [5] that was based in part on the estimation of 4-month-old breastfed infants’ free glutamate intake. A limitation of the present study is that we did not have similar measured outcomes of intake and body weight in a cohort of breastfed infants, and the glutamic acid content of their mothers’ milk over time, which would provide the gold standard in infant feeding.

Infants have been fed EHF since 1942, when the first infant formula for the nutritional management of cow milk allergy was launched, and these formulas have been evaluated for their suitability and safety in numerous preclinical and clinical studies. Most notably, the randomized controlled German Infant Nutrition Intervention study on infants who were at high risk for atopy revealed long-term benefits in preventing allergic outcomes 20 years after their last exposure to extensive protein hydrolysate formulas [10]. By implication, establishing an ADI of 30 mg/kg bw/day implies that feeding either human milk or infant formula may be unsafe and might induce alterations in neurodevelopment, but this is not the case. Furthermore, the global protein hydrolysate market is witnessing substantial growth [11] due to rising demands for protein-based dietary supplements and nutritional products, including infant and follow-on formulas [12, 13]. Protein hydrolysates, by their very nature, are high in free amino acids, including glutamic acid. Thus, we would predict that glutamic acid intake in those who consume hydrolysate-based products will be in excess of the recommended EFSA ADI.

The 2017 EFSA ADI was not based on actual intake data of the primary energy sources during infancy or on the established safety of these important sources of nutrition [5, 12]. It has been argued that nutritional recommendations on glutamic acid–glutamates for infants should not be based on an ADI for a food additive that is also an amino acid [6].The present findings, along with other scientific research on the growing child’s ingestion of glutamic acid from human milk, infant formula, and the complementary diet, may be of interest when developing communications to parents, clinical care providers, and policy makers as we all work to support nutritional health.

### Supplementary Information

Below is the link to the electronic supplementary material.

Supplementary Table 1 (online). Glutamic acid intake (mg/kg bw/d) from infant formula (A) and all other foods (B) and when combined (C, total) by infant formula group. Data are expressed as mean, median and lower and upper (25^th^, 75^th^) quartiles. (DOCX 19 KB)

Supplementary Table 2 (online): Enrollment (*n*) at each trial assessment. (DOCX 13 KB)

## Data Availability

Data described in the manuscript and codebook will be made available upon reasonable request and Data Usage Agreement sent to corresponding author, Dr. Mennella (mennella@monell.org).
