# Supplementary material for: Glutamic acid intake by formula-fed infants: are acceptable daily intakes appropriate?
Source: Eur J Pediatr. 2023 Sep 30;182(12):5701–5. doi: 10.1007/s00431-023-05215-6 (PMC10746772; doi:10.1007/s00431-023-05215-6)
Supplement: Supplementary file 1 — Supplementary Table 1 (online). Glutamic acid intake (mg/kg bw/d) from infant formula (A) and all other foods (B) and when combined (C, total) by infant formula group. Data are expressed as mean, median and lower and upper (25th, 75th) quartiles. (DOCX 19 KB) [file 431_2023_5215_MOESM1_ESM.docx]

| **Supplementary Table 1**. Glutamic acid intake (mg/kg bw/day) from infant formula (A) and all other foods (B) and when combined (C, total) by infant formula group. Data are expressed as mean, median and lower and upper (25^th^, 75^th^) quartiles. | | | | | | |
| --- | --- | --- | --- | --- | --- | --- |
|  | CMF Group | | | EHF Group | | |
| Infants’ Age (month) | Mean | Median | 25^th^, 75^th^ percentile | Mean | Median | 25^th^, 75^th^ percentile |
| A. Infant Formula | | | | | | |
| 0.5 | 544.4 | 510.5, | 436.3, 615.6 | 553.9 | 516.3 | 449.8, 608.3 |
| 0.75 | 502.5 | 507.0 | 434.5, 576.1 | 693.9 | 661.4 | 579.4, 840.1 |
| 1.5 | 587.3 | 578.3, | 461.1, 665.3 | 949.9 | 906.1 | 736.5, 1111.5 |
| 2.5 | 564.1 | 515.6 | 431.0, 664.7 | 964.5 | 879.3 | 710.7, 1152.9 |
| 3.5 | 432.5 | 408.4 | 349.8, 490.6 | 776.6 | 739.3 | 627.3, 934.0 |
| 4.5 | 465.9 | 428.6 | 371.3, 491.8 | 820.2 | 837.5 | 636.9, 998.1 |
| 5.5 | 434.5 | 399.7 | 353.9, 459.1 | 759.9 | 781.2 | 550.1, 901.7 |
| 6.5 | 384.7 | 374.0 | 302.7, 424.8 | 663.1 | 677.1 | 554.0, 816.6 |
| 7.5 | 313.2 | 285.5 | 239.8, 382.7 | 631.1 | 668.0 | 466.3, 778.7 |
| 8.5 | 312.2 | 279.5 | 226.2, 390.1 | 569.1 | 609.0 | 401.9, 706.2 |
| 9.5 | 274.8 | 244.3 | 210.9, 331.9 | 540.9 | 533.4 | 385.7, 651.8 |
| 10.5 | 276.8 | 252.2 | 184.4, 394.6 | 505.6 | 493.7 | 371.6, 621.8 |
| 11.5 | 233.9 | 242.6 | 172.3, 295.6 | 395.7 | 416.0 | 268.3, 543.7 |
| 12.5 | 183.3 | 206.7 | 128.1, 251.0 | 209.5 | 258.6 | 0, 349.0 |
| B. Other Foods | | | | | | |
| 0.5 | 0 | 0 | 0, 0 | 0 | 0 | 0, 0 |
| 0.75 | 0 | 0 | 0, 0 | 0.1 | 0 | 0, 0 |
| 1.5 | 5.1 | 0 | 0, 0 | 2.1 | 0 | 0, 0 |
| 2.5 | 3.5 | 0 | 0, 0 | 4.2 | 0 | 0,0 |
| 3.5 | 4.5 | 0 | 0, 0 | 7.5 | 0 | 0, 0 |
| 4.5 | 16.1 | 0 | 0, 16.1 | 28.4 | 0 | 0, 25.1 |
| 5.5 | 43.7 | 15.7 | 0, 63.7 | 47.2 | 17.9 | 0, 71.1 |
| 6.5 | 87.1 | 58.4 | 10.2,132.6 | 102.0 | 76.0 | 13.4, 128.7 |
| 7.5 | 123.6 | 107.4 | 60.8, 159.8 | 147.0 | 75.3 | 44.2, 201.8 |
| 8.5 | 177.7 | 89.1 | 65.6, 191.0 | 196.8 | 146.2 | 86.5, 187.0 |
| 9.5 | 265.4 | 218.7 | 69.1, 347.0 | 389.7 | 236.2 | 72.7, 566.2 |
| 10.5 | 333.3 | 236.0 | 113.1, 480.1 | 478.4 | 317.8 | 143.2, 639.7 |
| 11.5 | 444.0 | 390.4 | 266.7, 556.1 | 611.2 | 413.8 | 304.7, 937.5 |
| 12.5 | 593.4 | 598.6 | 389.6, 828.9 | 693.3 | 569.1 | 503.8, 986.9 |
| C. Total (Infant Formula + Other Foods) | | | | | | |
| 0.5 | 544.4 | 510.5 | 436.3, 615.6 | 553.9 | 516.3 | 449.8, 608.3 |
| 0.75 | 502.5 | 507.0 | 434.5, 576.1 | 694.0 | 661.4 | 579.4, 840.1 |
| 1.5 | 592.4 | 578.3 | 461.1, 665.3 | 952.0 | 906.1 | 736.5, 1111.5 |
| 2.5 | 567.6 | 520.7 | 431.0, 678.7 | 968.7 | 887.5 | 710.7, 1152.9 |
| 3.5 | 436.9 | 417.9 | 353.5, 499.0 | 784.1 | 753.1 | 627.3, 934.0 |
| 4.5 | 482.0 | 451.7 | 373.9, 532.2 | 848.5 | 845.2 | 686.3, 1023.0 |
| 5.5 | 478.1 | 442.6 | 393.5, 512.6 | 807.1 | 791.1 | 582.5, 970.5 |
| 6.5 | 474.4 | 474.8 | 366.0, 547.4 | 765.1 | 750.1 | 623.0, 897.4 |
| 7.5 | 436.8 | 402.2 | 343.7, 536.0 | 778.1 | 755.2 | 523.2, 913.0 |
| 8.5 | 489.9 | 393.8 | 312.6, 625.2 | 765.9 | 688.4 | 486.5, 1046.4 |
| 9.5 | 540.2 | 484.4 | 405.4, 616.2 | 930.6 | 806.2 | 539.6, 1243.0 |
| 10.5 | 610.1 | 568.2 | 332.8, 796.7 | 984.0 | 772.2 | 637.1, 1217.3 |
| 11.5 | 677.9 | 637.9 | 490.2, 858.9 | 1006.9 | 851.0 | 586.6, 1269.6 |
| 12.5 | 766.4 | 735.4 | 587.1, 945.1 | 896.1 | 925.5 | 639.4, 1111.2 |
| *Abbreviations*: CMF, cow milk formula; EHF, extensive protein hydrolysate formula | | | | | | |
