# Supplementary material for: Glutamic acid intake by formula-fed infants: are acceptable daily intakes appropriate?
Source: Eur J Pediatr. 2023 Sep 30;182(12):5701–5. doi: 10.1007/s00431-023-05215-6 (PMC10746772; doi:10.1007/s00431-023-05215-6)
Supplement: Supplementary file 2 — Supplementary Table 2 (online): Enrollment (n) at each trial assessment. (DOCX 13 KB) [file 431_2023_5215_MOESM2_ESM.docx]

| **Supplementary Table 2 (online)**: Enrollment (*n*) at each trial assessment | | | |
| --- | --- | --- | --- |
| Infants’ Age (months) | Both Groups | CMF Group | EHF Group |
| 0.5 | 113 | 59 | 54 |
| 0.7 | 113 | 59 | 54 |
| 1.5 | 109 | 57 | 52 |
| 2.5 | 107 | 55 | 52 |
| 3.5 | 101 | 53 | 48 |
| 4.5 | 92 | 48 | 44 |
| 5.5 | 90 | 46 | 44 |
| 6.5 | 90 | 46 | 44 |
| 7.5 | 89 | 46 | 43 |
| 8.5 | 89 | 46 | 43 |
| 9.5 | 87 | 45 | 42 |
| 10.5 | 85 | 45 | 40 |
| 11.5 | 84 | 44 | 40 |
| 12.5 | 83 | 44 | 39 |
| *Abbreviations*: CMF, cow milk formula; EHF, extensive protein hydrolysate formula | | | |
